# Temporal Dynamic Transcriptome Landscape Reveals Regulatory Network During the Early Differentiation of Female Strobilus Buds in *Ginkgo biloba*

**DOI:** 10.3389/fpls.2022.863330

**Published:** 2022-03-31

**Authors:** Pan-Pan Bai, Han-Yang Lin, Yue Sun, Jun-Jie Wu, Kai-Jie Gu, Yun-Peng Zhao

**Affiliations:** Laboratory of Systematic & Evolutionary Botany and Biodiversity, College of Life Sciences, Zhejiang University, Hangzhou, China

**Keywords:** flower bud differentiation, *Ginkgo biloba*, lncRNA, miRNA, ceRNA network

## Abstract

Reproductive bud differentiation is one of the most critical events for the reproductive success of seed plants. Yet, our understanding of genetic basis remains limited for the development of the reproductive organ of gymnosperms, namely, unisexual strobilus or cone, leaving its regulatory network largely unknown for strobilus bud differentiation. In this study, we analyzed the temporal dynamic landscapes of genes, long non-coding RNAs (lncRNAs), and microRNAs (miRNAs) during the early differentiation of female strobilus buds in *Ginkgo biloba* based on the whole transcriptome sequencing. Results suggested that the functions of three genes, i.e., *Gb_19790* (*GbFT*), *Gb_13989* (*GinNdly*), and *Gb_16301* (*AG*), were conserved in both angiosperms and gymnosperms at the initial differentiation stage. The expression of genes, lncRNAs, and miRNAs underwent substantial changes from the initial differentiation to the enlargement of ovule stalk primordia. Besides protein-coding genes, 364 lncRNAs and 15 miRNAs were determined to be functional. Moreover, a competing endogenous RNA (ceRNA) network comprising 10,248 lncRNA-miRNA-mRNA pairs was identified, which was highly correlated with the development of ovulate stalk primordia. Using the living fossil ginkgo as the study system, this study not only reveals the expression patterns of genes related to flowering but also provides novel insights into the regulatory networks of lncRNAs and miRNAs, especially the ceRNA network, paving the way for future studies concerning the underlying regulation mechanisms of strobilus bud differentiation.

## Introduction

A flower represents a unique feature of angiosperms, which is absent in its sister group, gymnosperms. Instead, the reproductive organ of gymnosperms, namely, the strobilus or cone, lacks the basic structure found in angiosperms and is more primitive than that of angiosperms. Compared with angiosperms, which are mostly bisexual, unisexual strobilus (dioecious or monoecious) is common among extant gymnosperm species. Of note, approximately 65% of gymnosperms are dioecious and 1% are monoecious (Walas et al., 2018), while only 6% of angiosperms are dioecious and 7% are monoecious (Diggle et al., 2011). The research focused on the differentiation of strobilus buds will lead to a comprehensive understanding of floral evolution as well as the determination and differentiation of unisexual reproductive organs.

Reproductive bud differentiation is one of the key stages in the life cycle of seed plants, which is regulated by both the intrinsic and the extrinsic factors (Fornara et al., 2010; Blumel et al., 2015; Cho et al., 2017). It comprises three phases, namely, the vegetative-to-reproductive transition phase, the organ patterning phase, and the organ development phase (Luo et al., 2013). The regulatory network of flower bud differentiation has been extensively studied in some model systems of angiosperms. The floral transition from the vegetative growth, i.e., floral induction, is mainly involved with six pathways in *Arabidopsis thaliana*, i.e., the vernalization pathway, the photoperiod pathway, the ambient temperature pathway, the age pathway, the autonomous pathway, and the gibberellin pathway (Fornara et al., 2010). The downstream integrators of the six upstream pathways rapidly promote floral development and activate meristem identity genes (Weigel et al., 1992; Moon et al., 2003; Navarro et al., 2011). Generally, A+E, A+B+E, B+C+E, C+E, and C+D+E control the formation of sepal, petal, stamen, carpel, and ovule, respectively (Theissen and Saedler, 2001). Besides protein-coding genes, two key microRNAs (miRNAs) (miR156 and miR172) acted as count-down and count-up timers in the floral induction, respectively (Aukerman and Sakai, 2003; Wu and Poethig, 2006; Chuck et al., 2007; Zhang et al., 2015). COOLAIR is the first well-characterized long non-coding RNA (lncRNA) that regulates vernalization by silencing *FLOWERING LOCUS C* (*FLC*) transcription transiently in *Arabidopsis* (Swiezewski et al., 2009). Recently, the competing endogenous RNA (ceRNA) hypothesis has gained increasing attention, which shows lncRNAs, pseudogenes, and circular RNAs (circRNAs) can act as ceRNAs to compete for binding miRNAs through miRNA response elements (MERs) and decrease the suppression of miRNAs to their target mRNA (Salmena et al., 2011). Two lncRNAs, i.e., XLOC_0063639 and XLOC_007072, were found to sequester miR160 or miR164 as ceRNAs in the sexual reproduction of rice (Zhang et al., 2014). A ceRNA network containing 78 lncRNAs, 397 miRNAs, and 32 mRNA was involved in the regulation of tomato flowering (Yang et al., 2019).

Compared with angiosperms, the genetic basis of strobilus bud differentiation in gymnosperms has been paid less attention. Nevertheless, previous studies have reported a series of homologous genes related to the differentiation of strobilus buds in gymnosperms. Homologous genes of *LEAFY* (*LFY*) have double-copy in gymnosperms, which suggested that they have undergone functional differentiation (Frohlich and Parker, 2000; Shindo et al., 2001; Dornelas and Rodriguez, 2005; Guo et al., 2005). *SUPPRESSOR OF OVEREXPRESSION OF CO 1*-like (*SOC1*-like) genes have been cloned in some gymnosperms, such as *Picea abies*, *Gnetum gnemon*, and *Pinus radiata* (Tandre et al., 1995; Walden et al., 1998; Winter et al., 1999). The BC/D model has been established in gymnosperms. The homeotic C and D class genes are used to distinguish reproductive organs from vegetative organs, while B class genes play a key role in specifying the identity of microsporophyll (Theissen et al., 2000). Yet, the regulatory network of strobilus bud differentiation remains unknown (Mao et al., 2019).

The living fossil ginkgo (*Ginkgo biloba* L.) is the sole living species of Ginkgophytes in gymnosperms whose female reproductive organ, i.e., the ovulate strobilus or the female cone, represents one of the most primitive compound strobilus types, making it of great importance in understanding the evolution of strobili and flowers (Fu and Yang, 1993). The differentiation process of gingko female strobilus buds (FSBs) consists of six stages, i.e., the vegetative growth, the initial differentiation, the exuberant differentiation, the integument differentiation, the nucellus differentiation, and the collar differentiation stages (Shi et al., 1998; Douglas et al., 2007). A total of 29 genes were determined to be closely related to three differentiation stages during the differentiation process (He et al., 2018). The homologous genes of *FLOWERING LOCUS T* (*FT*), *SOC1*, and *LFY* were cloned, respectively (Zhang et al., 2002; Guo et al., 2005; Wang et al., 2019; Feng et al., 2021). Of note, 11 and 37 MIKC*^c^*-type MADS-box genes were identified from a draft ginkgo genome and a new one, respectively (Guan et al., 2016; Chen et al., 2017; Liu et al., 2021). However, the functions of the homologous genes mentioned above have not been fully verified, and no attempts have been made to investigate the regulatory network of those genes or to pinpoint the role of non-coding RNAs (ncRNAs) during the differentiation of gingko FSBs.

In this study, we identified genes, lncRNAs, and miRNAs associated with the early differentiation of ginkgo FSBs using a whole-transcriptome sequencing strategy. We aimed (1) to explore the expression patterns of homologous genes (such as *FT*, *SOC1*, and *LFY*) related to the differentiation of ginkgo FSBs, (2) to identify the functional lncRNAs and miRNAs and draw the temporal dynamic expression landscapes, and (3) to explore the regulatory functions of ceRNA networks if exist. Our results provide new insights for further investigations on the regulatory networks of strobilus buds differentiation in gymnosperms, a land plant group with great economical, ecological, and evolutionary importance.

## Materials and Methods

### Plant Materials and Morphological Examination

Ginkgo FSBs were sampled from May to August in 2020 and 2021 from female trees with similar ages and growth conditions at Zhejiang University (Hangzhou, China) based on long-term observation. The fresh samples were fixed using 2.5% glutaraldehyde with the phosphate buffer (0.1 M, pH = 7.0). Post-fixation with 1% OsO_4_ in the phosphate buffer for 1–2 h precedes three times of washing for 15 min. Samples were subjected to gradient dehydration using ethanol solutions (30, 50, 70, 80, 90, and 95%, respectively) for 15 min and 100% ethanol twice for 20 min. Then, samples were dehydrated in a Hitachi Model HCP-2 critical point dryer (Hitachi, Tokyo, Japan) before being embedded with gold-palladium for 4–5 min in Hitachi E-1010 ion sputter (Hitachi, Tokyo, Japan). Later, we observed those samples using a Hitachi SU-8010 Scanning Electron Microscope (SEM) (Hitachi, Tokyo, Japan).

Based on the SEM observations, FSB samples for RNA sequencing were collected at three stages of strobilus buds each from three female trees according to the number and characteristics of ovulate stalk primordia in the FSBs (Shi et al., 1998; Douglas et al., 2007), i.e., the vegetative growth (ST1, sampled on May 6), the initial differentiation (ST2, sampled on May 26), and the exuberant differentiation (ST3). Given that the exuberant differentiation stage lasted for a long period, two samples with large morphological differences were sampled in each tree (ST3-1, sampled on June 19; ST3-2, sampled on July 28).

### Total RNA Extraction, Library Preparation, RNA Sequencing, and Quality Control

Total RNA was extracted using TRIzol Reagent (Invitrogen, Waltham, MA, United States) according to the manufacturer’s instructions. Subsequently, total RNA was qualified and quantified using NanoDrop ND-1000 (NanoDrop, Wilmington, DE, United States) and Agilent 2011 bioanalyzer (Agilent Technologies, Santa Clara, CA, United States).

For strand-specific libraries, approximately 1 μg of total RNA per sample was treated using the Ribo-Zero Plant Kit (Illumina, San Diego, CA, United States) to remove rRNA. The strand-specific libraries were constructed following RNA fragmentation, 1st cDNA strand synthesis, 2nd cDNA strand synthesis, end repair, tailing, and adaptor ligation. Finally, the qualified libraries were sequenced paired end on the Hiseq 4000 platform at BGI (Shenzhen, China). For small RNA libraries, approximately 1 μg of total RNA was used for each sample, and small RNA sequencing (sRNA-seq) was performed on the BGISEQ-500 platform (BGI, Shenzhen, China).

We removed adaptors, low quality, and ambiguous bases of raw data from strand-specific RNA-seq (ssRNA-seq) libraries and sRNA-seq libraries using SOAPnuke (Chen et al., 2018). Then, we discarded sRNA-seq reads shorter than 18 nt. After quality control using FastQC^1^, clean reads were used for the subsequent analyses.

### Reads Mapping and Transcript Quantification

The clean reads from ssRNA-seq were mapped to the updated draft ginkgo genome (Guan et al., 2019) using HISAT2 (Kim et al., 2019) with default parameters except “–rna-strandness RF.” StringTie (Pertea et al., 2015) was used to estimate the expression levels of all transcripts based on annotations of the ginkgo genome. The expression level of each gene was normalized to fragments per kilobase of transcript per million mapped reads (FPKM).

### Identification of lncRNAs

The mapped reads from ssRNA-seq were assembled using StringTie. Then, all StringTie assemblies were merged into a single transcriptome assembly using the StringTie merge operation. The GffCompare program (Pertea and Pertea, 2020) was used to compare merged transcripts and reference annotations. The LncRNAs were identified using the following steps: (1) transcripts were retained as the potential lncRNAs with the class codes of “u,” “i,” “x,” and “o”; (2) transcripts were retained with a length of >200 bp; (3) the coding potential of each transcript was estimated using the coding potential calculator (CPC) (Kang et al., 2017), coding-non-coding index (CNCI, version 2) (Sun et al., 2013), and PfamScan (Mistry et al., 2013). Transcripts were identified as lncRNAs if they were marked as “non-coding” by both CPC and CNCI as well as failed to map to the Pfam database. The expression level of each lncRNA was normalized to FPKM.

We identified putative target genes of lncRNAs by integrating differentially expressed lncRNAs (DElncRNAs) and differentially expressed genes (DEGs) in pairwise comparison among stages, i.e., ST1 vs. ST2, ST2 vs. ST3-1, and ST3-1 vs. ST3-2. According to the *cis*-regulation mechanism, lncRNAs can regulate their upstream and downstream genes. Thus, DEGs located in 10 kb upstream and downstream of DElncRNAs were treated as candidate target genes of DElncRNAs. In contrast, DEGs *trans*-regulation by DElncRNAs were predicted using lncTar (Li et al., 2015). The regulatory networks of lncRNA-mRNA were visualized using Cytoscape (Saito et al., 2012).

### Identification of miRNAs and miRNA Target Prediction

First, we converted clean reads from sRNA-seq into the required FASTA format of miRDeep-P2 (Kuang et al., 2019). Then, the FASTA files were mapped to the ginkgo genome using Bowtie (Langmead, 2010). The mapped reads were searched against the Rfam database (Kalvari et al., 2018; Kalvari et al., 2021) to remove rRNAs, tRNAs, snoRNAs, and snRNAs. The remaining reads were compared with conserved plant miRNAs in miRBase^2^ (Release 22.1) (Kozomara and Griffiths-Jones, 2014) using Bowtie with no more than one mismatch. The remaining reads were used to predict novel miRNAs using miRDeep-P2. The expression level of each miRNA was normalized to reads per million mapped reads (RPM). The targets of miRNAs were predicted using psRNATarget (Dai et al., 2018).

### Differential Expression Analysis and Function Enrichment Analysis

Differentially expressed genes, DElncRNAs, and differentially expressed miRNAs (DEmiRNAs) were identified by comparing two adjacent stages, i.e., ST1 vs. ST2, ST2 vs. ST3-1, and ST3-1 vs. ST3-2, with |log_2_FC| > 1 and adjusted *p*-value < 0.05 using the R package DESeq2 (Love et al., 2014). To indicate the expression patterns of DEGs and DElncRNAs, we grouped DEGs and DElncRNAs into different clusters using the R package Mfuzz (Kumar and Futschik, 2007).

The genome of ginkgo was functionally annotated using eggNOG (Huerta-Cepas et al., 2017). Gene ontology (GO) annotation was analyzed using the R package clusterProfiler (Yu et al., 2012) with a *p*-value of 0.05.

### Construction of ceRNA Regulatory Network

We constructed a lncRNA-miRNA-mRNA network based on the ceRNA hypothesis by integrating all DElncRNAs, DEmiRNAs, and DEGs in the ST2 vs. ST3-1 comparison as follows: (1) miRNA-lncRNA and miRNA-mRNA pairs were predicted using PsRNATarget with the default settings; (2) lncRNA-miRNA-mRNA pairs were integrated based on lncRNAs, and mRNAs were targeted by a common miRNA; and (3) the correlations of miRNA-lncRNA pairs and miRNA-mRNA pairs were evaluated based on the Spearman’s rank correlation coefficient (SCC) with ρ < -0.6. The Pearson correlation coefficient (PCC) was used to evaluate the strength of correlations between lncRNA-mRNA pairs, and pairs with *r* > 0.6 were selected. The lncRNA-miRNA-mRNA regulatory networks were visualized using Cytoscape.

## Results

### Morphology of Female Strobilus Buds During Early Differentiation

**FIGURE 1:**
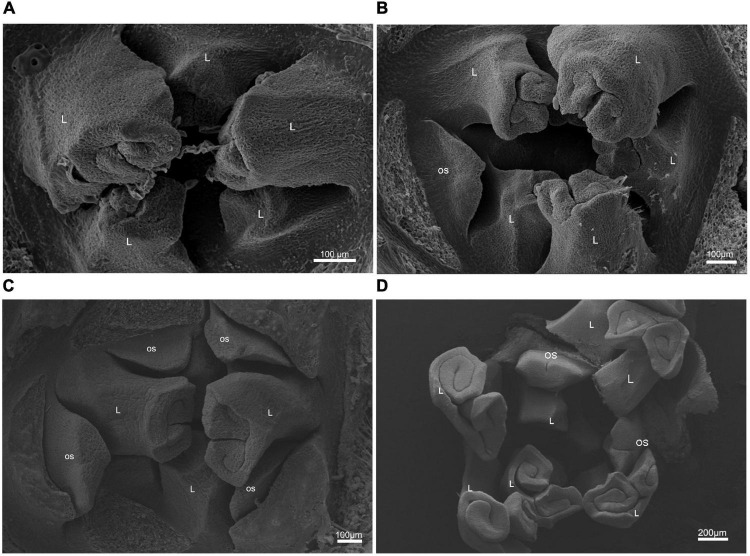
Morphology of the female strobilus buds of ginkgo at three developmental stages was observed using scanning electron microscopy (SEM). **(A)** At the vegetative stage (ST1), 3–7 leaf primordia curl inward in the buds but no ovule stalk appeared. **(B)** At the initial differentiation stage (ST2), an ovulate stalk primordium is initiated at the axils of a leaf primordium. **(C)** At the early exuberant differentiation stage (ST3-1), multiple ovulate stalk primordia appear. With the enlargement of ovulate stalk primordia, some of them take on frontally laminar shapes **(D)**. At the late exuberant differentiation stage (ST3-2), a longitudinal furrow becomes evident on the distal abaxial side of some ovulate stalk primordia. L, leaf primordium; OS, ovulate stalk primordium.

The differentiation process of ginkgo FSBs lasts nearly one year, which begins in May (for example, May 2020) and ends up in April of the following year (April 2021). This study focused on its early differentiation. The first stage of FSB differentiation was the vegetative stage when 3–7 leaf primordia curled inward in the buds before the end of May (ST1, Figure 1A). At the initial differentiation stage, an ovulate stalk primordium initiated at the axils of a leaf primordium or a bud bract, which was straighter than leaf primordia and in the form of a three-faced pyramid (ST2, Figure 1B). The subsequent stage is the exuberant differentiation stage (ST3), which was divided into two substages due to the long-lasting period from early July to early December. The first substage was characterized by the appearance and enlargement of multiple ovulate stalk primordia, and the ovulate stalk primordia were frontally laminar shape in appearance (ST3-1, Figure 1C). The second substage was featured by a longitudinal furrow appearing on the distal abaxial side of ovulated stalk primordium (ST3-2, Figure 1D).

### Expression Landscape of Genes During Early Differentiation of Female Strobilus Buds

**FIGURE 2:**
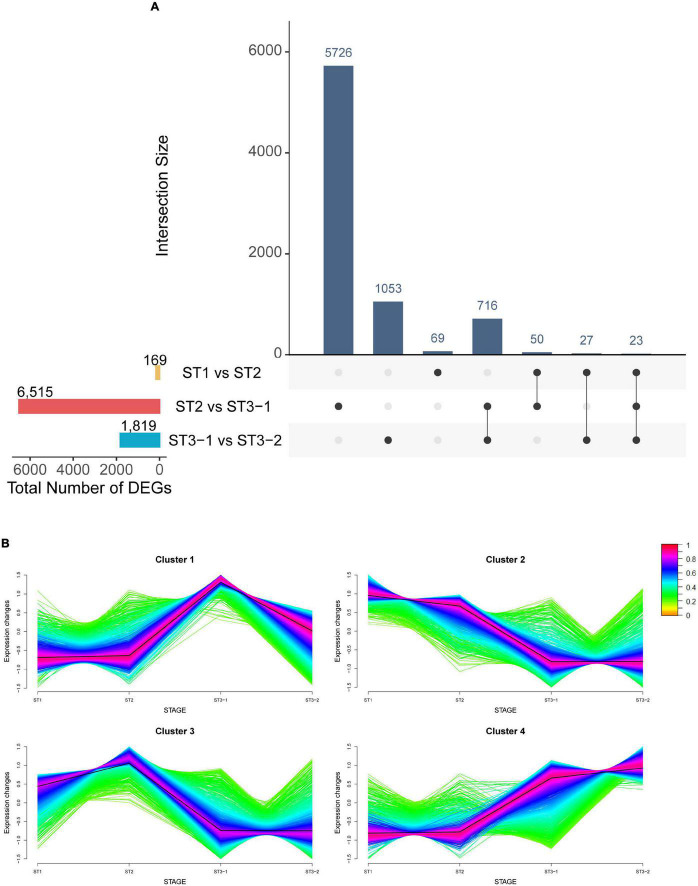
Characteristics of genes during the early differentiation of female strobilus buds in ginkgo. **(A)** The UpsetR plot shows the differentially expressed genes (DEGs) (|log_2_FC| > 1; adjusted *p*-value < 0.05) between the vegetative (ST1) and the initial differentiation (ST2) stages, the initial differentiation (ST2), and the early exuberant differentiation (ST3-1) stages, the early exuberant differentiation (ST3-1) and the late exuberant differentiation (ST3-2) stages. **(B)** The fuzzy C-means clustering identified four distinct temporal patterns of DEGs. The *x*-axis represents the differentiation stages of ginkgo female strobilus buds, while the *y*-axis represents the normalized expression changes in each stage.

Mapping clean reads from 12 strand-specific libraries to the ginkgo genome resulted in a 94.19–95.93% overall alignment rate. In total, 32,775 genes were expressed in at least one of the 12 samples. Furthermore, by comparing two adjacent stages (|log_2_FC| > 1 and adjusted *p*-value < 0.05), 8,503 DEGs were identified, including 169 DEGs between ST1 and ST2, 6,515 DEGs between ST2 and ST3-1, and 1,819 DEGs between ST3-1 and ST3-2, respectively (Figure 2A and Supplementary Table 1). The greatest difference in gene expression indicated that associated genes changed dramatically from the initial differentiation stage to the exuberant differentiation stage (Figure 2A).

The fuzzy C-means clustering identified four distinct temporal patterns of DEGs (Figure 2B). Clusters 2 and 4 represented downregulated and upregulated genes, respectively, while clusters 1 and 3 suggested that genes showed bimodal expression patterns. Interestingly, *Gb_19790* (*GbFT*) and *Gb_13989* (*LFY*-like) showed the highest expression level in ST2 and were clustered into cluster 3. Moreover, *Gb_16301* (*AGAMOUS*, *AG*) was also clustered into cluster 3, which plays a role in ovule identity and development (Pinyopich et al., 2003). *Gb_32549* (*CUP-SHAPED COTYLEDON 2*, *CUC2*) in cluster 4 was differentially expressed between ST2 and ST3-1, and the highest expression level was found in ST3-2.

Gene ontology analysis indicated that gene functions differed among the four clusters (Supplementary Table 2). The upregulated genes in cluster 4 fell into the categories of meristem development, meristem initiation, and meristem structural organization, among others. In contrast, the downregulated genes in cluster 2 were enriched in 32 categories, including response to triterpenoid metabolic process, isopentenyl diphosphate biosynthetic process, mevalonate pathway, and response to jasmonic acid. Genes in cluster 1 exhibited the highest expression level at ST3-1, which were involved in the process of mitochondrial RNA modification and metabolic processes. The expression level of genes in cluster 3 reached the highest point at ST2, and GO enrichment analysis of these genes showed that the top GO terms were response to heat, secondary metabolic process, and response to water deprivation.

### Expression Landscape of lncRNAs During Early Differentiation of Female Strobilus Buds

**FIGURE 3:**
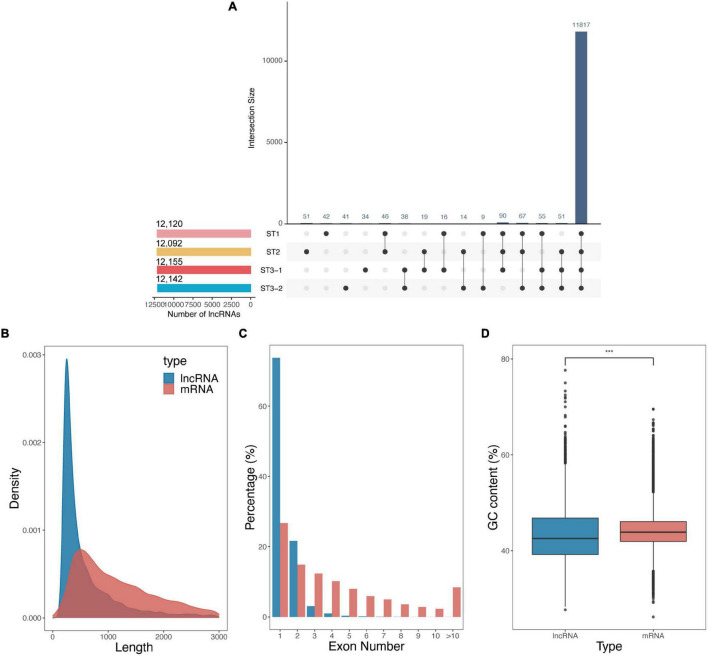
Characteristics of long non-coding RNAs (lncRNAs) during the early differentiation of female strobilus buds in ginkgo. **(A)** The UpsetR plot shows the number of lncRNAs identified from each stage, i.e., the vegetative (ST1), the initial differentiation (ST2), and the exuberant differentiation stages (ST3-1 and ST3-2). **(B–D)** Comparisons of lncRNAs and mRNAs with respect to the transcript length, exon number, and GC content.

Besides protein-coding genes, 12,408 lncRNAs were identified based on the transcript length, the type of class code, and the coding potential from 12 strand-specific libraries. Up to 95.2% of lncRNAs were continuously expressed during the early differentiation of FSBs, while only a few lncRNAs were stage-specific (Figure 3A). The average length of lncRNAs was shorter than that of mRNAs (801.30 nt vs. 1298.07 nt, Figure 3B). Meanwhile, lncRNAs contained strikingly fewer exons than mRNAs, i.e., one exon for 73.8% of lncRNAs vs. ≥ 2 exons for 73.3% of mRNAs (Figure 3C). The GC content of lncRNAs was significantly lower than that of mRNAs (*p*-value < 2.22e^–16^, Student’s *t*-test, Figure 3D). These results are consistent with previous studies on cucumber (He et al., 2020), poplar (Liu et al., 2019), and Chinese cabbage (Song et al., 2021).

Pairwise comparisons between the three developmental stages suggested that 12, 4,075, and 160 lncRNAs were differentially expressed for ST1 vs. ST2, ST2 vs. ST3-1, and ST3-1 vs. ST3-2 comparisons, respectively (Supplementary Table 3). The fuzzy C-means algorithm also clustered all of the DElncRNAs into four clusters (Supplementary Figure 1). These four clusters showed a similar pattern to that of DEGs, implying that DElncRNAs may play an important role in the differentiation process of FSBs.

**FIGURE 4:**
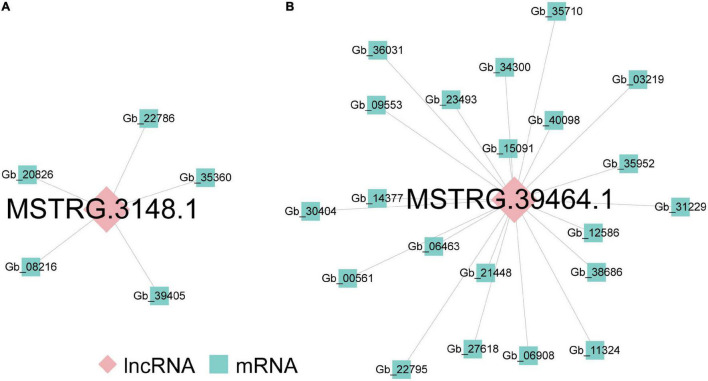
The lncRNA-mRNA co-expression networks during the initiation and enlargement of ovulate stalk primordia in ginkgo. **(A)** The lncRNA-mRNA co-expression network *trans*-regulated by differentially expressed lncRNAs (DElncRNAs) between the initial differentiation (ST2) and the early exuberant differentiation stages (ST3-1). **(B)** The lncRNA-mRNA co-expression network *trans*-regulated by DElncRNAs between the early (ST3-1) and the late (ST3-2) exuberant differentiation stages.

Long non-coding RNAs often mediate transcriptional regulation *via cis*- or *trans*-regulation (Rinn and Chang, 2012). As for *cis*-regulated analysis, 380 lncRNA-mRNA pairs, including 308 DEGs located 10 kb upstream and downstream of 351 DElncRNAs, composed the colocation network of the ST2 vs. ST3-1 comparison (Supplementary Figure 2). GO analysis showed that these DEGs were related to photosynthesis (Supplementary Table 4). In addition, 11 lncRNA-mRNA pairs, including 11 DElncRNAs and nine DEGs, formed the colocation network of ST3-1 vs. ST3-2 comparison (Supplementary Figure 3). Genes in this colocation network were significantly enriched in six GO categories, such as organic hydroxy compound biosynthetic process, saponin metabolic process, and glycoside biosynthetic process (Supplementary Table 4). In contrast, lncRNAs work in *trans*-regulation when they modulate genes across multiple chromosomes (Kornienko et al., 2013). We found that MSTRG.3148.1 modulated five DEGs in the ST2 vs. ST3-1 comparison by *trans*-regulation (Figure 4A), and MSTRG.39464.1 *trans*-regulated 21 DEGs, which composed a co-expression network in the ST3-1 vs. ST3-2 comparison (Figure 4B). However, no lncRNA was detected in regulating protein-coding genes through *cis*- and *trans*-regulation in the ST1 vs. ST2 comparison.

### Expression Landscape of miRNAs During Early Differentiation of Female Strobilus Buds

**FIGURE 5:**
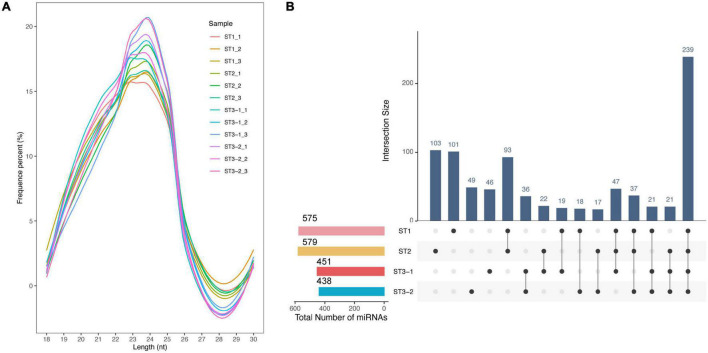
Characteristics of microRNAs (miRNAs) during the early differentiation of female strobilus buds in ginkgo. **(A)** Length distribution of the mappable sRNA reads from 12 libraries. **(B)** The UpsetR plot shows the number of miRNAs identified from each stage, i.e., the vegetative (ST1), the initial differentiation (ST2), and the exuberant differentiation stages (ST3-1 and ST3-2).

The most mapped clean reads are 24 nt in length from 12 small RNA libraries (Figure 5A). We identified 398 conserved and 471 novel miRNAs totally. Most miRNAs were stage-specific, while only 239 miRNAs were expressed in all samples (Figure 5B), 57 DEmiRNAs (| log_2_FC| > 1 and adjusted *p*-value < 0.05), including 11, 48, and 5 DEmiRNAs in the ST1 vs. ST2, ST2 vs. ST3-1, and ST2 vs. ST3-1 comparisons, respectively (Supplementary Table 5). Such a difference supported a remarkable transition from the initial differentiation to the early exuberant differentiation stage, in which miRNAs might play a role.

**FIGURE 6:**
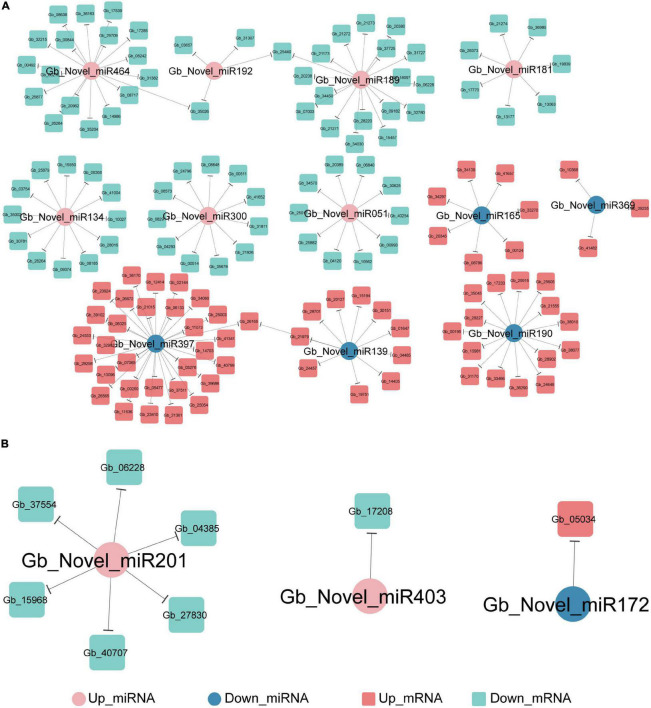
The miRNA-mRNA regulatory networks during the initiation and enlargement of ovulate stalk primordia in ginkgo. **(A)** The target of genes of differentially expressed miRNAs (DEmiRNAs) between the initial differentiation (ST2) and the early exuberant differentiation stages (ST3-1). **(B)** The target of genes of differentially expressed miRNAs (DEmiRNAs) between the early (ST3-1) and the late (ST3-2) exuberant differentiation stages.

PsRNATarget analysis predicted that 144 DEGs were negatively targeted by 12 novel DEmiRNAs in the ST2 vs. ST3-1 comparison (Figure 6A) and eight DEGs were targeted by three novel DEmiRNAs in the ST3-1 vs. ST3-2 comparison (Figure 6B), while none of them were found in the ST1 vs. ST2 comparison. In the ST2 vs. ST3-1 comparison, three novel miRNAs, i.e., Gb_Novel_miR397, Gb_Novel_miR464, and Gb_Novel_miR189, were predicted to target 33, 18, and 18 genes, respectively. GO analysis showed that three genes (*Gb_21271*/*Gb_21272*/*Gb_21273*) targeted by Gb_Novel_miR189 and *Gb_26565* targeted by Gb_Novel_miR397 were enriched in the GO term of positive regulation of cellular amide metabolic process. In the ST3-1 vs. ST3-2 comparison, Gb_Novel_miR331 might target six genes. However, target genes of known miRNAs were not found in this study.

### Long Non-coding RNA-Associated ceRNA Network Related to the Initiation and Enlargement of Ovulate Stalk Primordium

**FIGURE 7:**
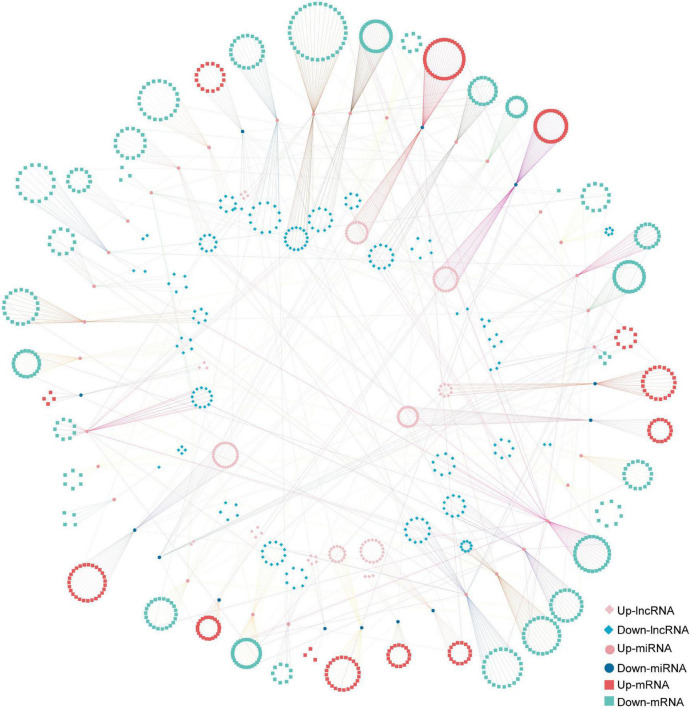
The long non-coding RNA-associated competing endogenous RNA (ceRNA) network between the early (ST3-1) and the late (ST3-2) exuberant differentiation stages in ginkgo.

**FIGURE 8:**
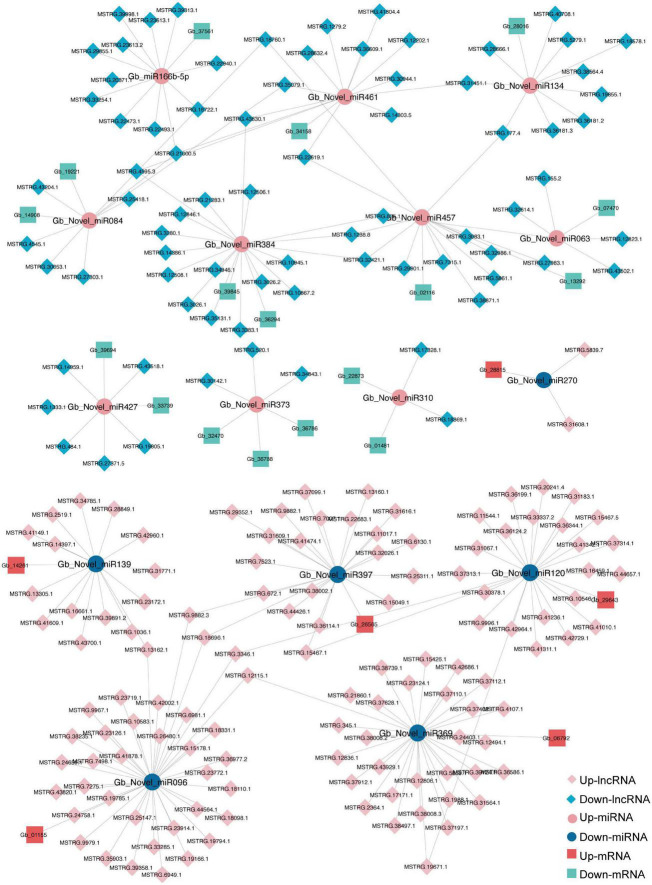
A sub-competing endogenous RNA (ceRNA) network is involved in GO term of response to water between the early (ST3-1) and the late (ST3-2) exuberant differentiation stages in ginkgo.

The ceRNA network based on DElncRNAs, DEmiRNAs, and DEGs in the ST3-1 vs. ST3-2 comparison predicted one lncRNA-miRNA-mRNA pair, i.e., MSTRG.2203.13-Gb_Novel_miR190- *Gb_35828*. In addition, 10,247 lncRNA-miRNA-mRNA pairs, which contained 1,225 nodes (393 lncRNAs, 44 miRNAs, and 788 mRNAs) and 1,361 edges (miRNA-target pairs), were found in the ST2 vs. ST3-1 comparison (Figure 7 and Supplementary Table 6). A total of three novel miRNAs, i.e., Gb_Novel_miR369, Gb_Novel_miR096, and Gb_Novel_miR397, were involved in most ceRNA pairs, suggesting that they may act as core regulators in the initiation and enlargement of ovulate stalk primordium in ginkgo. Of note, 13 lncRNAs were found to act as ceRNAs to compete for binding Gb_miR166b-5p and regulated the expression of 22 genes. The ceRNA pairs regulated by Gb_miR535-5p included three lncRNAs and four mRNAs. GO analysis showed that 23 genes that responded to water were regulated by 16 DEmiRNAs and 200 DElncRNAs (Figure 8). These results demonstrated the potential function of the ceRNA network during the initiation and enlargement of ovulate stalk primordium in ginkgo.

## Discussion

### Protein-Coding Genes Are Dominant When the Differentiation of Female Strobilus Buds Starts

Two “flowering-time” genes, i.e., *FT* and *SOC1*, act as integrators in the regulatory network for the timing of floral transition in *Arabidopsis*. *GbFT* was verified to promote vegetative-to-reproductive induction (Wang et al., 2019), which is also supported in this study. The expression level of *Gb_19790* (*GbFT*) was the highest at the initial differentiation stage and decreased along with the differentiation of strobilus buds. In contrast, *SOC1*, a member of the MIKC-type MADS-box genes, was named *GbMADS6* (*Gb_01884*) and is believed to promote the transition of the vegetative shoot meristem into the reproductive meristem in ginkgo (Feng et al., 2021). In this study, the trend of its expression is the same as that of Feng et al. (2021), though *GbMADS6* was not differentially expressed between the vegetative (early May) and the initial differentiation stages (end of May). The function of *GbMADS6* needs to be further studied in future works.

*LEAFY* patterns the reproductive meristem by regulating *APETALA3* (*AP3*, B function) and *AGAMOUS* (*AG*, C function) genes in angiosperms (Moyroud et al., 2011) and is conserved in gymnosperms and angiosperms (Moyroud et al., 2010). The two paralogous *LFY*-like genes in ginkgo, i.e., *Gb_05859* (*GinLfy*) and *Gb_13989* (*GinNdly*), showed different expression patterns that the expression level of *GinNdly* reached the highest at the initial differentiation stage, whereas *GinLfy* did not express differently in every pairwise comparison. We proposed that *GinNdly* was related to the development of FBSs in ginkgo, which is consistent with a previous study (Guo et al., 2005). The expression of *WeILFY* and/or *WeINDLY* always precedes that of *WeIAP3*/*PI*, and there is strong *WeIGA* expression in the male tissues of *Welwitschia* (Moyroud et al., 2017). Here, the expression pattern of *Gb_16301* (*AG*) was the same as that of *GinNdly* (Figure 2B). Therefore, we proposed that controlling the *AG*-like MADS-box gene by *NDLY* homologous predated the origin of the female reproductive tissues in ginkgo.

Besides protein-coding genes, we identified 12,120 and 12,092 lncRNAs in the vegetative and the initial differentiation stages, respectively (Figure 3A). COOLAIR, a cold-induced *FLC* antisense transcript, played a role in early cold-induced silencing of *FLC* transcription (Swiezewski et al., 2009). However, we failed to predict COOLAIR, which may result from the FLC subfamily in MIKC-type genes having been lost in ginkgo (Chen et al., 2017; Liu et al., 2021). There were 12 lncRNAs and 11 miRNAs differently expressed, but their putative target genes were not predicted from our results. We proposed that protein-coding genes are dominant when the differentiation of ginkgo FSB starts.

### Both Genes and Non-coding RNAs Are Involved in the Initiation and Enlargement of Ovulate Stalk Primordia

The greatest expression differences of genes, lncRNAs, and miRNAs were found between the initial differentiation and the early exuberant differentiation stages, i.e., 6,515 DEGs, 4,075 DElncRNAs, and 48 DEmiRNAs (Supplementary Tables 1, 3, 5), which implied that the largest changes occurred during the initiation and the enlargement of the ovulate stalk primordium. Of note, 1,819 DEGs, 160 DElncRNAs, and five DEmiRNAs were found between the early and late differentiation stages (Supplementary Tables 1, 3, 5). CUC proteins, the members of the NAC (NAM/ATAF/CUC) family, play important roles as boundary-defining factors during plant development in *Arabidopsis* (Ishida et al., 2000; Goncalves et al., 2015). *Gb_32549* (*CUC2*) was upregulated at the early exuberant differentiation stage, which indicated that the expression of *Gb_32549* can predict the boundary of the ovulated stalk primordium in ginkgo.

As for lncRNAs, *cis*- and *trans*-regulated analyses indicated that 362 and two DElncRNAs mediated the transcriptional regulation in the initiation and the enlargement of ovulate stalk primordia, respectively. The *cis*-regulating pairs were predominant in lncRNA-mRNA pairs here, which indicated that lncRNAs mainly regulated their target genes by *cis*-regulating in ginkgo (Supplementary Figures 2, 3). GO analysis showed that genes targeted by DElncRNAs had diverse functions (Supplementary Table 4). Three genes (*Gb_09034*, *Gb_09035*, and *Gb_26289*) *cis*-regulated by five lncRNAs were significantly enriched in the GO term of chlorophyll-binding. *Gb_11209* and *Gb_32759* were related to the biosynthetic and metabolic of the organic hydroxy compound and were *cis*-regulated by MSTRG.39998.1 and MSTRG.39391.1, respectively. In contrast, MSTRG.3148.1 and MSTRG.39464.1 functioned through *trans*-regulation and targeted five and 21 genes, respectively (Figure 4). Nine conserved miRNA families were related to flower development in plants. In this study, eight (miR156, miR159, miR160, miR166/165, miR167, miR169, miR172, and miR319) of them were identified (Luo et al., 2013), and 15 novel DEmiRNAs were predicted to target 152 DEGs (Figure 6). Gb_Novel_miR397 can target the greatest number of genes among DEmiRNAs, suggesting its essential roles in the initiation and enlargement of ovulate stalk primordia.

### LncRNAs Act as ceRNAs Participating in the Initiation and Enlargement of Ovulate Stalk Primordia

A lncRNA-associated ceRNA network comprised of 10,248 lncRNA-miRNA-mRNA pairs was determined to be associated with the initiation and enlargement of ovulate stalk primordia (Figure 7 and Supplementary Table 6). Some genes and miRNAs were proven to regulate the development of flower buds and ovules previously. ARGONAUTE10 (AGO10) functions as a decoy for miR166/165 to regulate *HD-ZIP III* genes in SAM development and maintenance in *Arabidopsis* (Zhu et al., 2011). However, none of the genes targeted by Gb_miR166b-5p belongs to the *HD-ZIP III* transcription factors in this study. The function of Gb_miR166b-5p remains to be determined. MiR535 negatively regulated rice immune responses and cold tolerance in rice (Sun et al., 2020; Zhang et al., 2022); four genes (*Gb_07340*, *Gb_10299*, *Gb_18723*, *Gb_30145*) and three lncRNAs (MSTRG.14303.4, MSTRG.14303.9, MSTRG.23460.1) were predicted by Gb_miR535-5p. Water is confirmed to be related to the initiation and enlargement of ovule primordia in angiosperms (Clepet et al., 2021). Here, a sub-ceRNA network related to water consists of 200 lncRNAs, 16 miRNAs, and 23 genes. The ceRNA network may add more layers of complexity in the regulatory network of initiation and enlargement of ovulate stalk primordia.

## Conclusion

As the first attempt, this study performed a genome-wide analysis to determine the roles of genes, lncRNAs, and miRNAs during the early differentiation of ginkgo FSBs. The temporal dynamic expression landscapes of genes and ncRNAs suggest that (1) the functions of some genes are conserved in both gymnosperms and angiosperms, such as *GbFT* (*FT*), *GinNdly* (*LFY*-like), Gb*_16301*(*AG*), and *Gb_32549* (*CUC2*); (2) apart from genes, lncRNAs and miRNAs are closely associated with the dramatic morphological changes during the initiation and enlargement of ovulate stalk primordia; and (3) lncRNAs play vital roles as ceRNAs during the differentiation of flower buds. To conclude, this study provides key candidate transcripts related to the differentiation of strobilus buds for further wet-bench experiments in gymnosperms.

## Data Availability Statement

The datasets presented in this study can be found in online repositories. The names of the repository and accession number can be found below: GinkgoDB with the accession number T0001 (https://ginkgo.zju.edu.cn/project/T0001/).

## Author Contributions

Y-PZ conceived the ideas and supervised this project. P-PB, YS, and H-YL collected the samples and performed the experiments. P-PB conducted data analysis. P-PB and H-YL wrote the manuscript with the contributions from all co-authors. All authors read and approved the final manuscript.

## Conflict of Interest

The authors declare that the research was conducted in the absence of any commercial or financial relationships that could be construed as a potential conflict of interest.

## Publisher’s Note

All claims expressed in this article are solely those of the authors and do not necessarily represent those of their affiliated organizations, or those of the publisher, the editors and the reviewers. Any product that may be evaluated in this article, or claim that may be made by its manufacturer, is not guaranteed or endorsed by the publisher.

## Footnotes

1
http://www.bioinformatics.babraham.ac.uk/projects/fastqc/

2
http://www.mirbase.org/
