# Supplementary material for: Temporal Dynamic Transcriptome Landscape Reveals Regulatory Network During the Early Differentiation of Female Strobilus Buds in Ginkgo biloba
Source: Front Plant Sci. 2022 Mar 31;13:863330. doi: 10.3389/fpls.2022.863330 (PMC9008512; doi:10.3389/fpls.2022.863330)
Supplement: Supplementary file 1 [file Data_Sheet_1.docx]

Supplementary Material

# Supplementary Figures


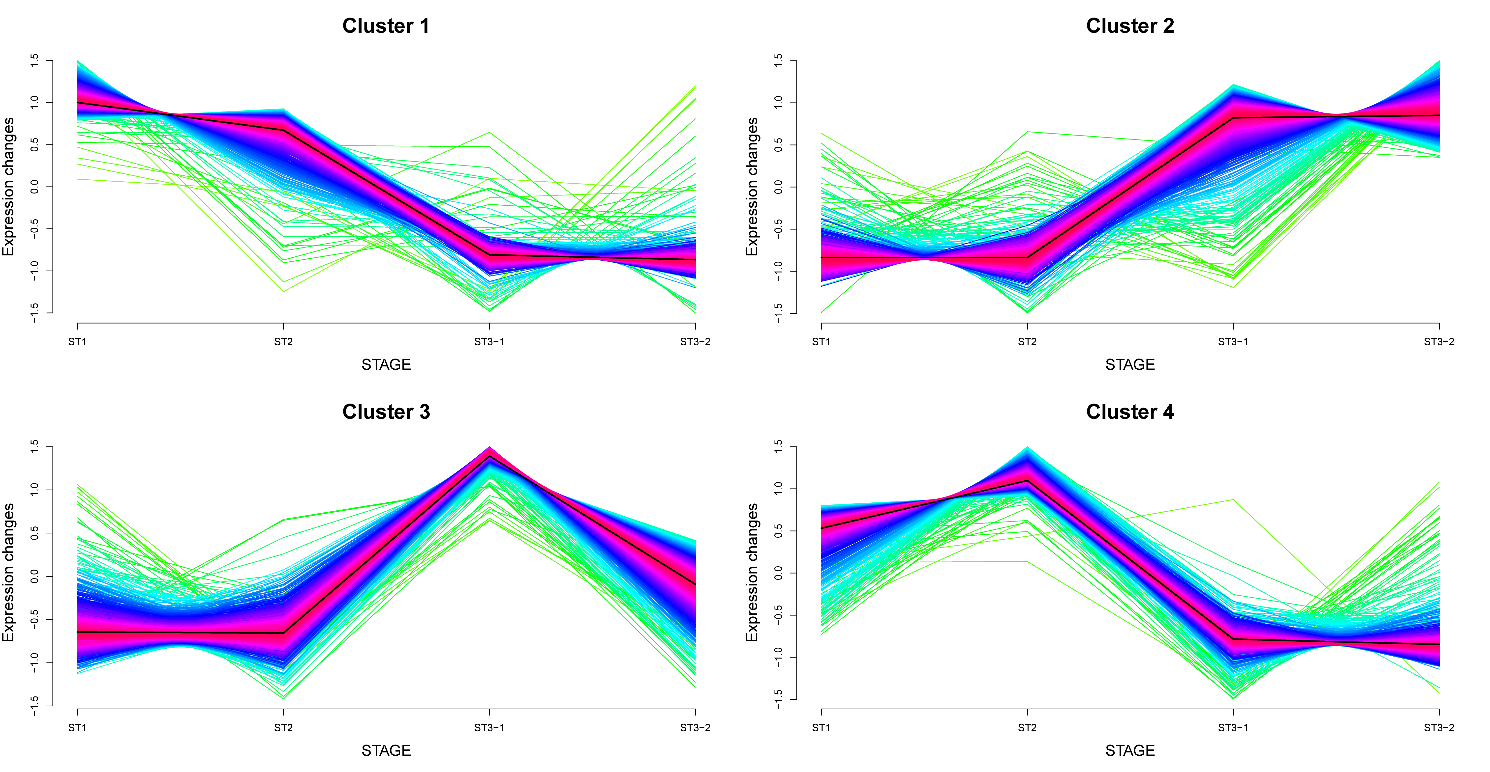


**Supplementary Figure 1.** The fuzzy C-means clustering identified four distinct temporal patterns of differentially expressed long non-coding RNAs (DElncRNAs). The *x*-axis represents the differentiation stages of ginkgo female strobilus buds, while the *y*-axis represents the normalized expression changes in each stage.


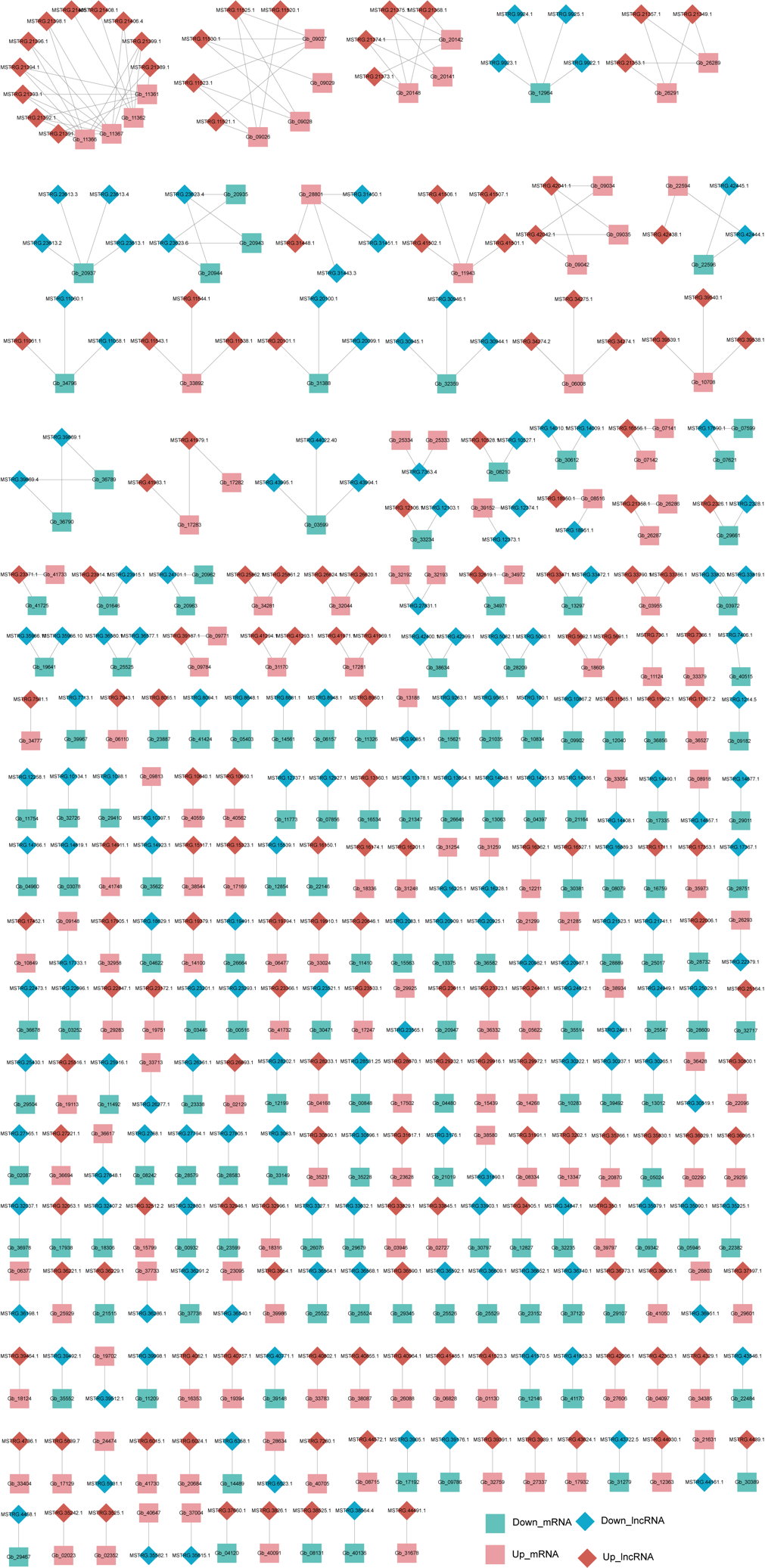


**Supplementary Figure 2.** The co-location network *cis*-regulated by differentially expressed lncRNAs (DElncRNAs) between the initial differentiation (ST2) and the early exuberant differentiation stages (ST3-1)


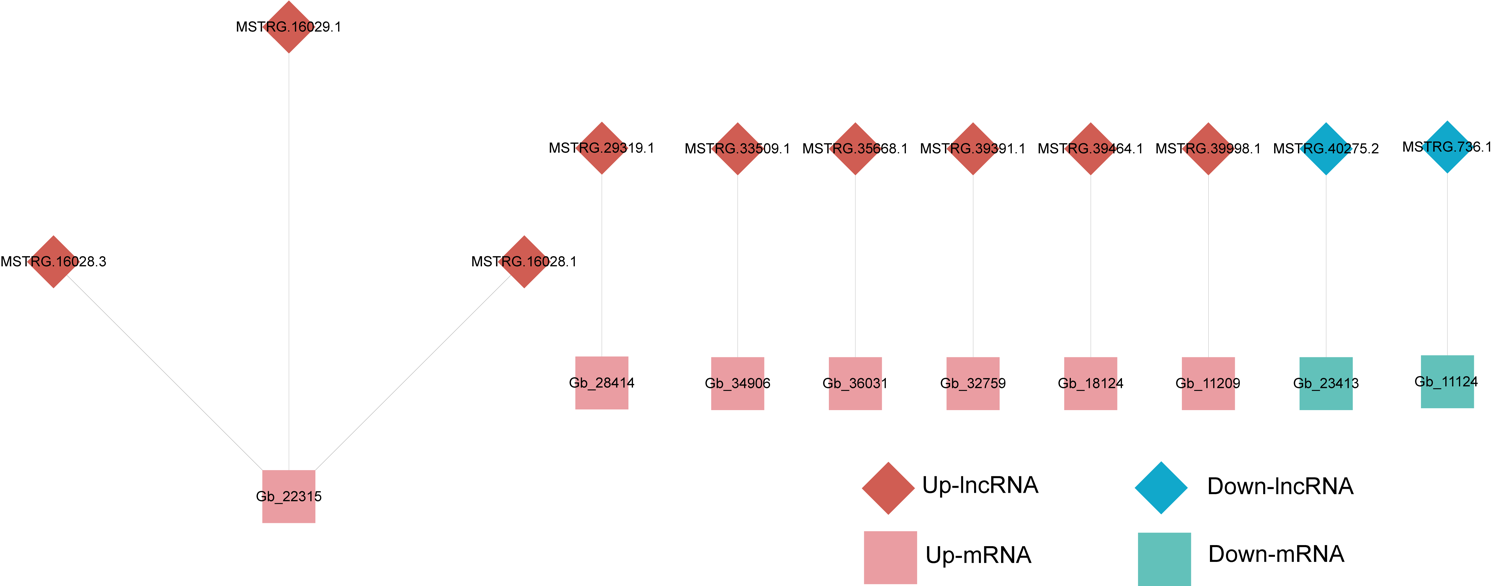


**Supplementary Figure 3.** The lncRNA-mRNA co-location network *cis*-regulated by differentially expressed lncRNAs (DElncRNAs) between the early (ST3-1) and the late (ST3-2) exuberant differentiation stages.
